# Hydrothermal Modification of Activated Carbon Enhances Acetaminophen Adsorption: Experimental and Computational Evidence of π–π Interaction Dominance

**DOI:** 10.3390/molecules30214295

**Published:** 2025-11-05

**Authors:** Astrid G. Cortés-Cruz, Marta Adame-Pereira, Carlos J. Durán-Valle, Ignacio M. López-Coca

**Affiliations:** 1 Área Académica de Química, Universidad Autónoma del Estado de Hidalgo, Mineral de la Reforma C.P. 42184, Mexico; co483027@uaeh.edu.mx; 2 Departamento de Química Orgánica e Inorgánica, Universidad de Extremadura, Avda. del Elvas, s/n, 06006 Badajoz, Spain; martaap@unex.es (M.A.-P.);; 3 Instituto Universitario del Agua, el Cambio Climático y la Sostenibilidad, Universidad de Extremadura, Avda. del Elvas, s/n, 06006 Badajoz, Spain; 4 Instituto Universitario para el Desarrollo Territorial Sostenible, Universidad de Extremadura, Avda. de la Universidad, s/n, 10003 Cáceres, Spain

**Keywords:** activated carbon, hydrothermal treatment, acetaminophen/paracetamol adsorption, pharmaceutical contaminants, DFT modeling, π–π interactions

## Abstract

Acetaminophen (APAP) is a widely used pharmaceutical increasingly detected as a contaminant in aquatic environments due to its persistent nature and incomplete removal by conventional wastewater treatment. This study investigates the adsorption performance and mechanisms of commercial activated carbon (M) and its hydrothermally modified form (MH) for APAP removal. Characterization via elemental analysis, X-ray photoelectron spectroscopy (XPS), and N_2_ adsorption isotherms revealed that hydrothermal treatment reduced oxygen content and enhanced micro- and mesopore volumes, resulting in a more homogeneous and carbon-rich surface. Batch adsorption experiments conducted under varying pH (5–7) and temperature (30–40 °C) conditions showed that MH achieved up to 94.3% APAP removal, outperforming the untreated carbon by more than 15%. Kinetic modeling indicated that adsorption followed a pseudo-second-order mechanism (R^2^ > 0.99), and isotherm data fitted best to the Langmuir model for MH and the Freundlich model for M, reflecting their differing surface properties. Adsorption was enhanced at lower pH and higher temperatures, consistent with an endothermic and pH-dependent mechanism. Complementary density functional theory (DFT) simulations confirmed that π–π stacking is the dominant interaction between APAP and the carbon surface. The most favorable configuration involved coplanar stacking with non-oxidized graphene (ΔG = −33 kJ/mol), while oxidized graphene models exhibited weaker interactions. Natural Bond Orbital (NBO) analysis further supported the prevalence of π–π interactions over dipole interactions. These findings suggest that surface deoxygenation and improved pore architecture achieved via hydrothermal treatment significantly enhance APAP adsorption, offering a scalable strategy for pharmaceutical pollutant removal in water treatment applications.

## 1. Introduction

Acetaminophen (APAP), also known as paracetamol or N-(4-hydroxyphenyl)acetamide, is a widely used over-the-counter and prescription non-steroidal analgesic and antipyretic. Its primary pharmacological action involves the inhibition of prostaglandin synthesis through cyclooxygenase (COX-1 and COX-2) pathways, thereby alleviating pain and reducing fever. Global consumption of APAP is substantial, estimated at nearly 1.5 tons annually [1,2,3]. China and India currently lead global production, accounting for over 70% of the total supply [4,5].

Despite its therapeutic benefits, APAP has been increasingly recognized as a persistent and ubiquitous contaminant in aquatic environments, posing a pressing environmental and health concern. Its widespread usage, combined with incomplete removal in conventional wastewater treatment plants (WWTPs), contributes to its frequent detection in wastewater effluents, surface waters, groundwater, and even seawater. Reported concentrations range from nanograms to milligrams per liter [1,6,7]. With a solubility of 305 mg/L at 25 °C and a Log K_ow_ of 0.46, APAP is relatively hydrophilic and poorly adsorbed onto organic matter, contributing to its mobility in aquatic systems. Following administration, approximately 90% of APAP is excreted via urine in both unchanged and conjugated forms [8]. Additional pathways of environmental contamination include discharge from pharmaceutical manufacturing and improper disposal of expired or unused medications [1]. The direct release of untreated or partially treated wastewater, a significant source of APAP, can have severe ecotoxic effects on aquatic species and carcinogenic and mutagenic effects on humans [9].

The environmental occurrence of APAP has consequences. It has been documented in surface water, groundwater, and drinking water sources across 29 countries, with reported concentrations averaging 0.161 μg/L and reaching a maximum of up to 230 μg/L. These concentrations, even at the lower end of the range, are significant and indicate the widespread presence of APAP in our water sources, posing potential risks to both the environment and human health [10].

Human health risks associated with APAP include severe hepatotoxicity, oxidative stress, DNA damage, and liver failure in overdose scenarios. Environmentally, it is classified as a high-risk pollutant, particularly to aquatic organisms [11,12,13]. Aquatic invertebrates such as *Daphnia magna*, as well as fish species like *Danio rerio* and trout, exhibit sensitivity to APAP even at trace levels, manifesting in oxidative stress, enzyme inhibition, and developmental impairments [14,15]. Notably, paracetamol was identified as the NSAID (nonsteroidal anti-inflammatory drugs) that disrupted the most significant number of oxidative stress biomarkers, underscoring its ecotoxicological relevance [16]. Plants also experience phytotoxic effects, including reduced concentrations of photosynthetic pigments and inhibited root growth [13].

While the challenges are significant, the potential of advanced treatment solutions beyond conventional methods is promising. A recent survey of 20 WWTPs in China revealed APAP in 19 of the samples, with concentrations ranging from 0.06 to 29.2 nM. However, despite high removal rates reported for APAP during secondary treatment [3,17,18], more toxic metabolites such as *p*-aminophenol have been detected post treatment at elevated concentrations (23.93–108.68 nM), indicating the existence of metabolic bottlenecks in existing treatment processes [19]. This highlights the need for advanced treatment solutions beyond conventional methods, along with continuous monitoring strategies to mitigate potential environmental and health risks [8,20,21].

Several advanced remediation technologies have been explored for the removal of APAP. Among these, Advanced Oxidation Processes (AOPs) have garnered considerable interest due to their ability to generate highly reactive radicals, particularly hydroxyl (•OH), capable of mineralizing organic pollutants into benign by-products [3,22]. In AOPs, hydroxyl radicals play a key role in breaking down organic pollutants, including APAP, into simpler, less harmful compounds, thereby reducing their environmental impact. However, AOPs face limitations, including incomplete mineralization and the formation of toxic intermediates—especially during photocatalytic oxidation. Additionally, UV-based systems are energy-intensive, and their efficiency can be diminished by background organic matter or radical-scavenging ions present in complex water matrices. Some of these problems are being solved using techniques such as cold plasma treatment [23], which allows organic contaminants to be eliminated with lower energy consumption and at ambient temperature and pressure. Membrane technologies, including microfiltration, ultrafiltration, nanofiltration, and reverse osmosis, provide an advanced treatment pathway. While these systems offer high separation efficiencies, they are often hindered by membrane fouling, increased energy consumption, and high operational costs, particularly in pressure-driven applications such as reverse osmosis [24,25]. Additionally, high energy consumption is a notable drawback, particularly in pressure-driven systems, such as reverse osmosis, which require substantial energy inputs to maintain optimal performance. Another problem with membrane filtration is that its results depend on the size of the molecules [26]. Although it can be a suitable method for separating contaminants when they have some value, its results may not be optimal for complex matrices.

Electrochemical methods, particularly Electrochemical Advanced Oxidation Processes (EAOPs), provide a flexible alternative for degrading pharmaceutical pollutants through redox reactions [27]. Recent innovations in nanostructured electrodes and catalytic coatings have significantly improved performance [28]. Integration with biological and membrane-based technologies has also demonstrated synergistic potential in complex treatment scenarios.

Biological remediation approaches, including phytoremediation and microbial biodegradation, have shown promise due to their sustainability and cost-effectiveness. Phytoremediation relies on specific plant species—often hardy or invasive—to absorb and transform pharmaceuticals through enzymatic pathways [29,30]. Similarly, microbial biodegradation utilizes bacteria, fungi, and microalgae capable of enzymatically degrading APAP via pathways involving amidases, oxygenases, and deaminases [13]. The low operational cost and environmental compatibility of biological methods make them an attractive option for sustainable water treatment. However, these methods are sensitive to environmental factors such as temperature, pH, and the presence of co-contaminants, which can impair microbial activity and overall efficiency.

Among the various remediation technologies, adsorption has emerged as one of the most promising strategies for removing emerging contaminants, including pharmaceutical contaminants from water systems [31,32,33,34,35]. Adsorbents, such as activated carbon and biochar, capitalize on their high surface area, porosity, and tunable surface chemistries to effectively capture organic contaminants [36]. Activated carbon, available in both granular (GAC) and powdered (PAC) forms, is particularly valued for its cost-effectiveness, chemical stability, and high adsorption capacity. Surface modifications, including oxidation and amination, further enhance its performance by introducing reactive functional groups that improve affinity toward ionizable or polar compounds such as APAP [20,37,38,39]. Advanced materials such as metal–organic frameworks (MOFs), carbon nanotubes (CNTs), and graphene derivatives exhibit even higher adsorption capacities. However, their high production costs and limited scalability remain significant barriers to widespread application [40,41]. In contrast, activated carbon offers a practical and scalable solution, especially for large-scale water treatment applications. Many of the methods mentioned above (e.g., AOPs) have a clear disadvantage compared to adsorption: although they can be very effective against organic pollutants, they do not usually act on inorganic compounds. Given that in most situations, wastewater contains a complex mixture of both types of products, it is logical to conclude that adsorption should be a widely used option. Despite its advantages, adsorption-based remediation is not without challenges. Regeneration of spent adsorbents via thermal or chemical means is often energy-intensive and can degrade material integrity over time [42]. Moreover, safe disposal or recycling of exhausted adsorbents is critical to prevent secondary contamination. Nonetheless, continued advancements in adsorbent materials and system design underscore the viability of adsorption, particularly using activated carbon, as a cornerstone strategy for APAP remediation.

Enhancing the yield and performance of activated carbons can be effectively achieved through modifications to their surface chemistry and porous structure. In this context, we have explored hydrothermal modification as a viable strategy to improve the physicochemical properties of activated carbon. This approach has yielded promising results in previous studies, where the modified materials demonstrated high adsorption capacities for contaminants such as thorium [43], uranium [44], and acetamiprid [45]. Additionally, these materials have shown catalytic activity in reactions relevant to the synthesis of fine chemicals [46], further highlighting their versatility and potential for multifunctional applications.

In this context, the present study explores the use of activated carbon for the adsorption and removal of acetaminophen from contaminated water. Particular emphasis is placed on elucidating the adsorption mechanisms, evaluating key performance parameters, and assessing the practical feasibility of applying this approach in real-world water treatment systems.

## 2. Results and Discussion

### 2.1. Characterization of Carbonaceous Materials

The proximate analysis (heating program detailed in the Supplementary Materials) yielded comparable results for both materials, with a slight reduction in fixed carbon content and a corresponding increase in volatile matter in the hydrothermally treated sample (MH), as shown in Table 1. Both samples exhibit a high fixed carbon content, indicative of a significant degree of carbonization, characteristic of activated carbons.

The elemental composition of the organic phase (excluding ash content) is summarized in Table 2. Hydrothermal treatment results in an increase in carbon, nitrogen, and sulfur content, and a reduction in hydrogen and oxygen. The former three elements are typically embedded within the graphene basal plane and thus exhibit lower reactivity. Conversely, hydrogen and oxygen, commonly located at the edges or defects of the carbon matrix, are more susceptible to removal under hydrothermal conditions.

X-ray photoelectron spectroscopy (XPS) further confirms these trends. Atomic and mass-based compositions are reported in Table 3 and Table 4, respectively. Both samples display surfaces enriched in carbon, with MH showing a more uniform composition and detectable nitrogen content, which was not detected in M.

**Figure 1:**
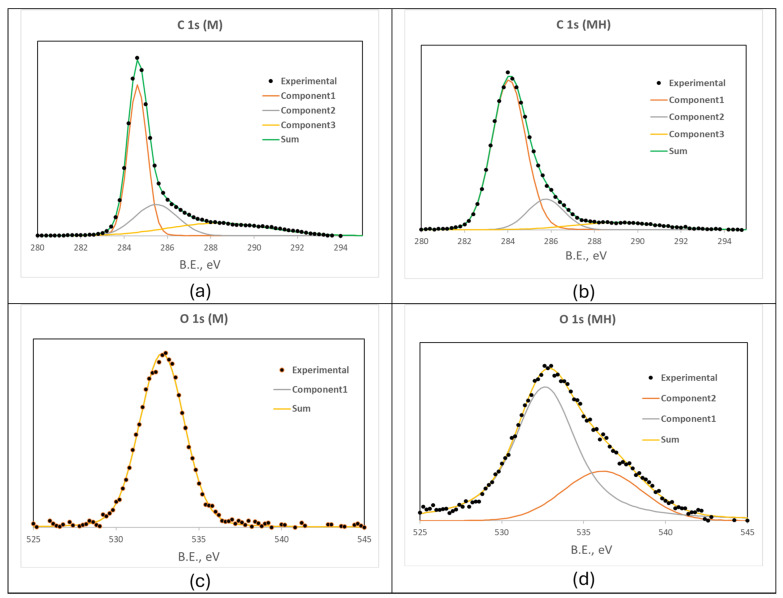
C 1s XPS spectra for (**a**) M and (**b**) MH. O 1s XPS spectra for (**c**) M and (**d**) MH.

Deconvolution of the C 1s peak (Figure 1, Table 5) reveals that hydrothermal treatment significantly reduces the proportion of moderately oxidized carbon species (285.7 to 286.5 eV) and strongly oxidized carbon species (288.6 to 289.6 eV), associated with hydroxyl/ether/carbonyl and carboxylic/ester groups, respectively. In contrast, the signal at 284.8 eV, corresponding to sp^2^ carbon or C–H/C–C bonding, increases in MH.

The deconvolution of the O 1s peak, while limited due to overlap and complexity of functional groups, reveals the emergence of a secondary component at 536.8 eV in MH in addition to the only one shown by M (533 eV), suggesting structural rearrangements during treatment (Figure 1). Other elemental peaks (as N 1s) did not present sufficient resolution for further analysis.

The point of zero charge (PZC) increased from 7.51 (M) to 8.06 (MH), indicating a reduction in surface acidity. This is consistent with the XPS results showing a decrease in oxygenated groups following hydrothermal treatment. It is known [47] that a higher oxygen content usually leads to higher acidity in carbonaceous materials. In our case, the component that can be assigned to carboxyl groups in the C 1 s spectrum (288.6/289.6 eV) is lower in MH coal, making it more alkaline. This basicity stems mainly from the high density of delocalized π-electrons in the basal planes of carbon [48]. Other functional groups (e.g., sulfonic groups) could indeed produce greater acidity with lower oxygen content [49], but this is not the case with these coals, as they do not contain sulfur.

**Figure 2:**
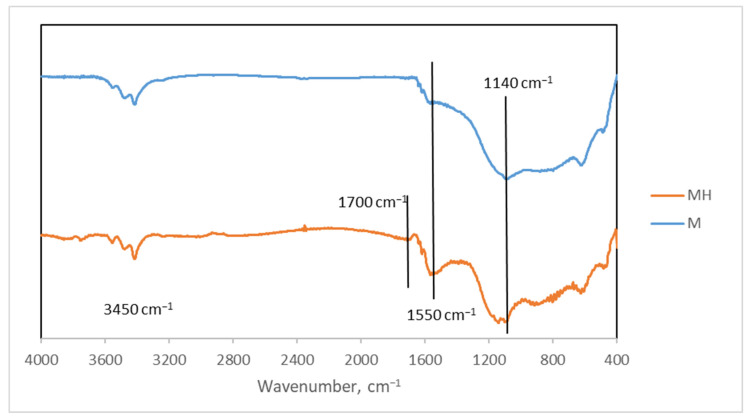
FTIR spectra of M and MH.

The FTIR spectra of both samples is shown in Figure 2. There are some slight differences between them.

The bands near 3450 cm^−1^ (O-H) are similar, so this functional group must continue to exist without significant changes. However, the band at 1700 cm^−1^ (C=O) only appears in MH, so the carbonyl groups (in small proportions, as it is a weak band) must be formed during hydrothermal treatment. There is also an increase in the band at 1550 cm^−1^ (C=C) when compared to the area between 1500 and 1300 cm^−1^, so we must assume changes in that part of the structure corresponding to carbon backbones. Changes are also observed in the band corresponding to C-O bonds (near 1140 cm^−1^), as there is a maximum in M but a double peak in MH. This corroborates what is shown in the O 1s spectrum of XPS (Figure 1c,d), where noticeable changes have been observed.

**Figure 3:**
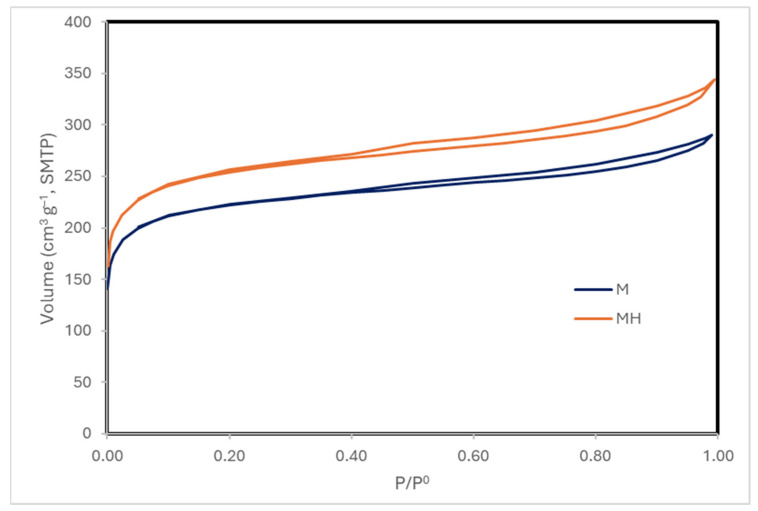
N_2_ adsorption isotherms at 77 K.

Nitrogen adsorption–desorption isotherms (Figure 3) demonstrate that both samples exhibit type IV isotherms with H4 hysteresis loops [50], typical of micro-mesoporous carbonaceous materials. MH displays a higher total N_2_ uptake.

**Figure 4:**
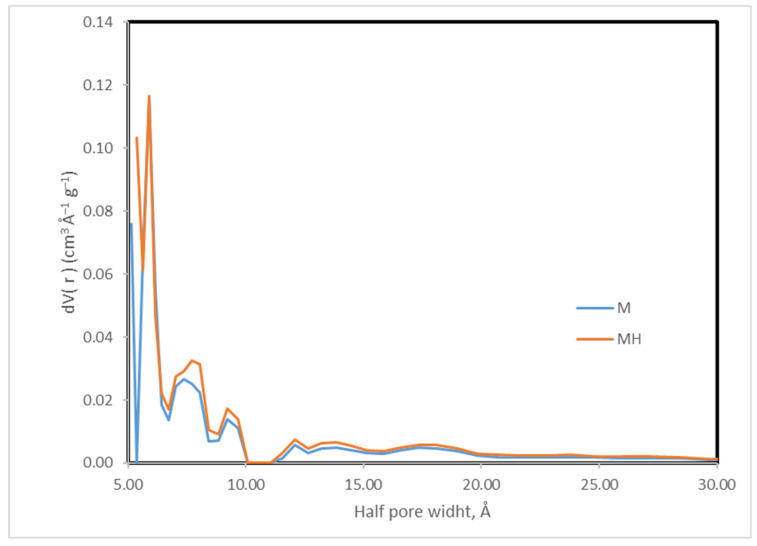
Pore size distribution (DFT method).

Although MH shows a slight decrease in surface area, it exhibits higher micropore and mesopore volumes, which contribute to its improved adsorption performance. The pore size distribution (Table 6, Figure 4) further indicates a broader and slightly larger distribution in MH. In contrast, SEM micrographs (Figures S1 and S2, Supplementary Information) do not reveal any substantial morphological alterations. Possible changes in the crystallinity of the samples were also compared using X-ray diffraction (Figure S5), but no significant variations were found.

### 2.2. Adsorption Experiments

The adsorption kinetics of acetaminophen (APAP) were evaluated using both adsorbent materials, M and MH, under varying pH (5, 6, and 7) and temperature conditions (30 °C and 40 °C). These parameters were selected to simulate hospital wastewater discharge scenarios, where APAP concentrations might be elevated. At these pH levels, APAP (pK_a_ = 9.5) [51] predominantly exists in its neutral molecular form.

**Figure 5:**
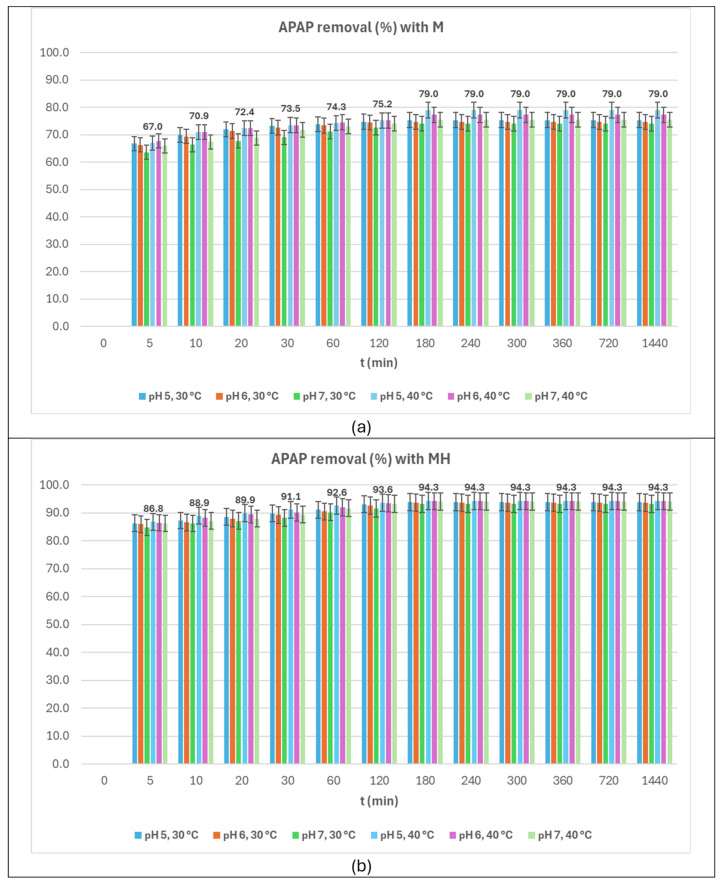
APAP removal kinetics for (**a**) M and (**b**) MH. Numeric data from pH 5, 40 °C experiment.

As shown in Figure 5a,b, the adsorption equilibrium was reached within 180 min for both adsorbents. MH consistently exhibited a significantly higher adsorption capacity than M across all conditions, with differences exceeding 15 percentage points. This enhanced performance is attributed to improved pore accessibility and surface chemistry introduced by hydrothermal treatment, which likely increases the availability of active sites. These conditions facilitate stronger adsorbate–adsorbent interactions, such as, π–π stacking, particularly relevant at pH values where APAP remains neutral. The sharper initial removal rates for MH suggest reduced diffusion resistance and improved mass transport properties.

To elucidate the adsorption mechanism, the kinetic data were fitted to pseudo-first-order and pseudo-second-order models:

- Pseudo-first-order model:

(1) $$log\left(q_{e}-q_{t}\right)=\log q_{e}-\frac{k_{1}}{2.303}t$$

- Pseudo-second-order model:

(2) $$\frac{t}{q_{t}}=\frac{1}{k_{2}^{2}q_{e}^{2}}+\frac{1}{q_{e}}t$$

where *q_t_* is the amount of adsorbate adsorbed at time *t* (mg/g); *q_e_* is the amount of adsorbate adsorbed in equilibrium; *k*_1_ and *k*_2_ are the kinetic constant of the models (pseudo-first order: min^−1^, pseudo-second-order: g/mg min); *t* is the time (min).

The pseudo-second-order model provided a significantly better fit across all experimental conditions (R^2^ > 0.99). For example, under the MH_40_pH5 condition, an R^2^ of 0.9995 was obtained, with a rate constant *k* = 0.2413 min^−1^ and a calculated equilibrium removal of 94.3%. The kinetic parameters obtained are shown in Table 7. Plots showing the variation in concentration over time can be found in the Supplementary Materials (Figure S4).

**Figure 6:**
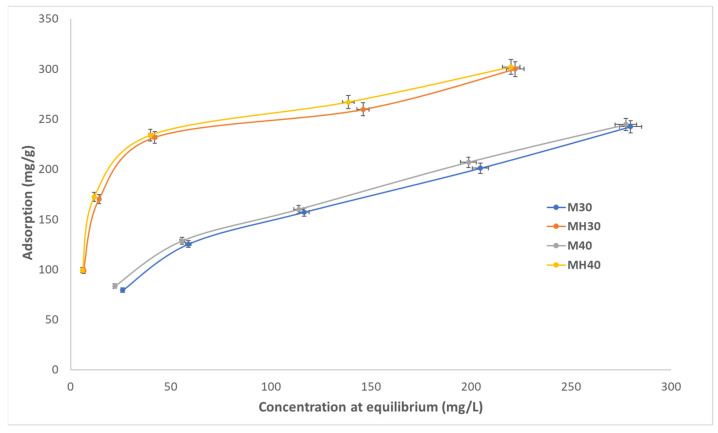
Adsorption isotherm for APAP onto M and MH at pH 5, 30 °C and 40 °C.

**Figure 7:**
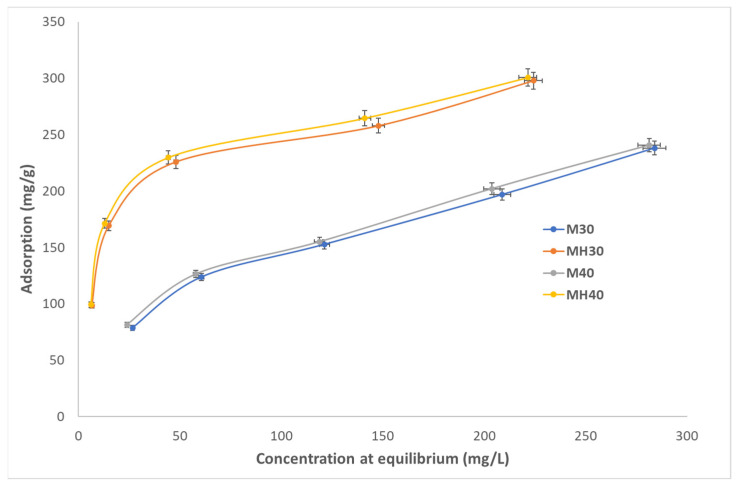
Adsorption isotherm for APAP onto M and MH at pH 6, 30 °C and 40 °C.

**Figure 8:**
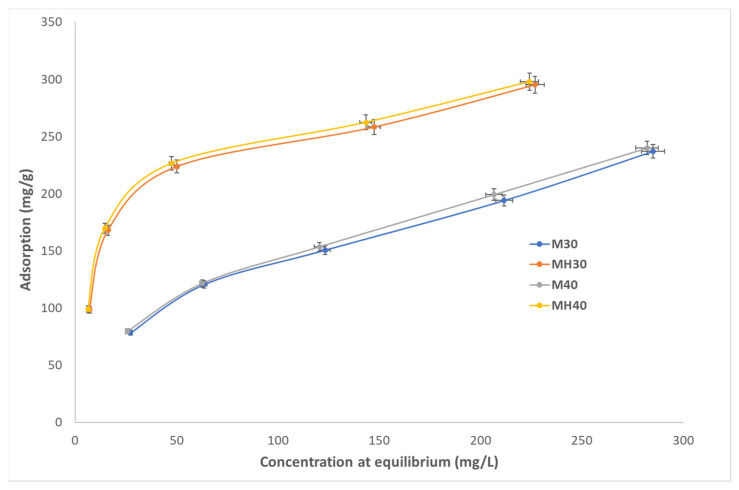
Adsorption isotherm for APAP onto M and MH at pH 7, 30 °C, and 40 °C.

Equilibrium adsorption isotherms were obtained at pH 5, 6, and 7, and at 30 °C and 40 °C for both materials (Figure 6, Figure 7 and Figure 8). MH consistently outperformed M, with adsorption efficiency increasing by up to 19.9%. The calculated q_e_ values are similar to the experimental values in the pseudo-second-order model, demonstrating the validity of this model compared to the pseudo-first-order model. This result confirms the significant difference in the value of R^2^ between the two models. The results of adsorption obtained at a temperature of 40 °C are higher than those obtained at 30 °C, which can be explained by the fact that a higher temperature increases the mobility of the molecules, speeding up diffusion and promoting adsorption. However, pH has a lesser influence, although it has been observed that an increase in pH results in processes with lower adsorption efficiency. According to the calculated k values, they do not necessarily have to be slower.

The equilibrium data were modeled using the Langmuir and the Freundlich isotherms (Table 8):

- Langmuir equation:

(3) $$\frac{C_{e}}{q_{e}}=\frac{1}{K_{L}S_{m}}+\frac{C_{e}}{S_{m}}$$

where *K_L_* is the Langmuir constant and *S_m_* is the adsorption capacity of a monolayer on the adsorbent.

- Freundlich equation:

(4) $$\log q_{e}=\log K_{F}+\frac{1}{n}\log C_{e}$$

where *K_F_* is the Freundlich constant that increases with the capacity of the adsorbent.

Carbon M fits the Freundlich model more closely, suggesting a heterogeneous surface with multiple active site types. DFT simulations (see below) show that oxygenated functional groups can exhibit a wide range of adsorption energies. Since carbon M has a greater number of these groups, a more heterogeneous surface is to be expected. In contrast, MH adheres better to the Langmuir model, indicating a more uniform surface chemistry and monolayer adsorption behavior. Despite similar monolayer capacities, experimental adsorption was consistently higher for MH. The Freundlich parameter *n*, which reflects adsorption favorability, was lower for MH, yet experimental results contradicted this, further supporting that different materials may align with different mechanistic models. As they respond to different mechanisms, the comparison of these parameters may not be correct.

To assess performance at higher concentrations, the effect of initial APAP concentration was evaluated. Table 9 summarizes these results. At low concentrations (100 mg/L), MH removed >93% of APAP across all conditions. Even at 500 mg/L, MH maintained a removal efficiency > 57%, significantly outperforming M.

In conclusion, MH exhibits superior adsorption performance primarily due to changes in textural and chemical properties induced by hydrothermal treatment. The influence of temperature and pH, while present, is comparatively secondary. Adsorption is slightly more favorable at lower pH due to speciation effects and is enhanced at higher temperatures, suggesting an endothermic mechanism.

### 2.3. Computational Study

To gain further insight into the adsorptive interactions between APAP and the carbonaceous materials, a computational study was conducted using graphene and oxidized graphene models (see Supplementary Information, Figure S3). Geometry optimization was performed as outlined in the Experimental |Section. The oxidized graphene model included representative hydroxyl, carbonyl, and carboxylic functional groups.

**Figure 9:**
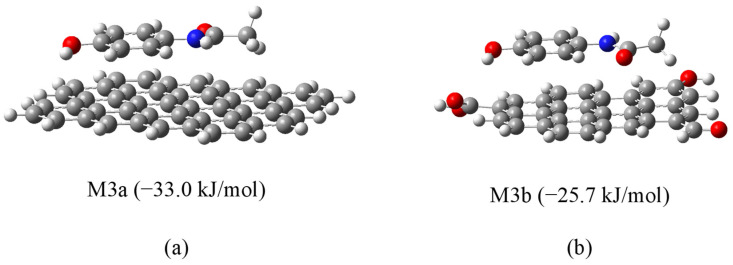
Optimized geometries of APAP interaction with (**a**) non-oxidized (M3a) and (**b**) oxidized graphene (M3b).

The most stable configuration (model M3a) corresponded to a coplanar π–π stacking interaction between APAP and non-oxidized graphene, with a calculated free energy of adsorption of −33.0 kJ/mol (Figure 9a). In comparison, the analogous interaction with oxidized graphene (model M3b) yielded a less favorable ΔG of −25.7 kJ/mol (Figure 9b). This result is consistent with experimental findings, in which MH (lower oxygen content) exhibited higher adsorption capacity than M. However, it is noteworthy that this trend is not universal; for instance, prior simulations with phenol demonstrated enhanced adsorption on oxidized graphene [52].

**Figure 10:**
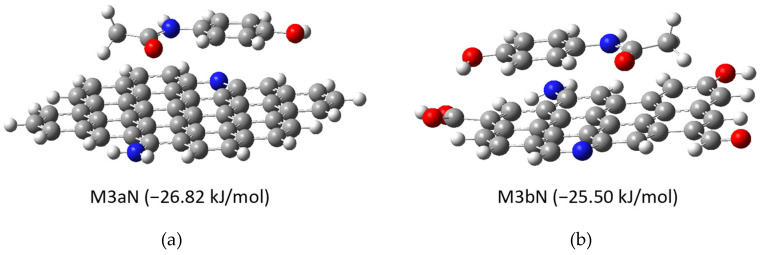
Optimized geometries of APAP interaction with (**a**) non-oxidized and nitrogenated (M3aN) and (**b**) oxidized and nitrogenated graphene (M3bN).

Given the lack of reliable data (XPS) on the nitrogen present in M and MH carbons, we have performed other simulations in which nitrogen atoms are added to the graphene molecule (models M2aN and M2bN, see Supplementary Materials) as well as simulating the interaction with APAP (models M3aN and M3bN, Figure 10).

The presence of heteroatoms (N and O) reduces the calculated interaction energy. The joint existence of N and O in the graphene molecule (M3bN) does not seem to offer a significant variation compared to the presence of only one of the two elements (M3b and M3aN).

**Figure 11:**
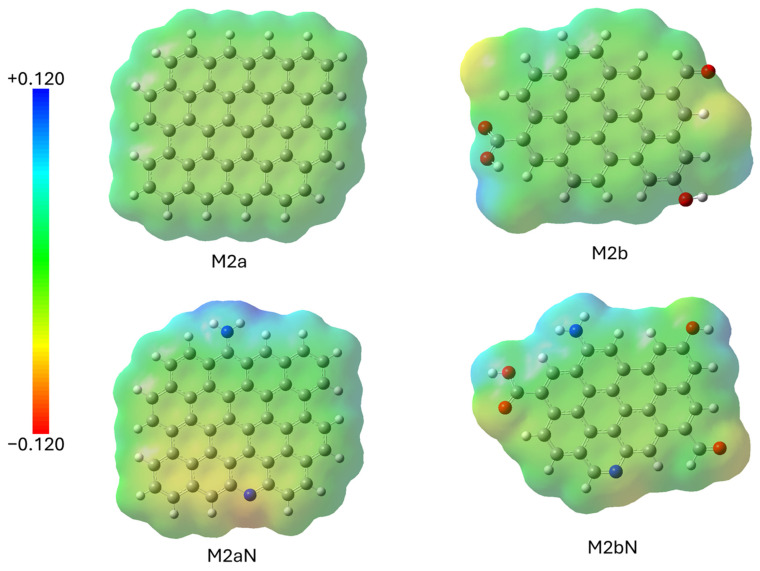
Electronic distribution in different graphene models.

The study of the electronic distribution in the graphene molecule (Figure 11) shows that the presence of heteroatoms distorts the uniformity observed in M2a, creating negatively charged areas (mainly near O and N) and positively charged areas (mainly near H atoms bonded to O or N).

Natural Bond Orbital (NBO) analysis revealed four significant interactions (>0.5 kcal/mol) in M3a, M3aN, and M3bN versus two in M3b. These were primarily π–π stacking interactions, although two donor–acceptor interactions between APAP hydroxyl groups and graphene aromatic carbons are present as well. The stability of M3a suggests that π–π stacking dominates the adsorption mechanism.

**Figure 12:**
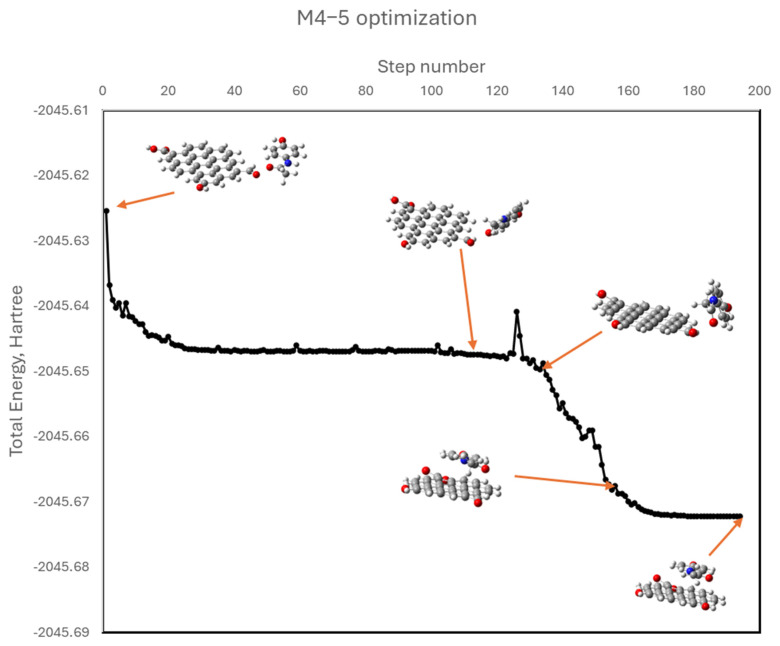
Optimization trajectory of model M4-5 showing APAP shifting into π–π stacked conformation.

**Figure 13:**
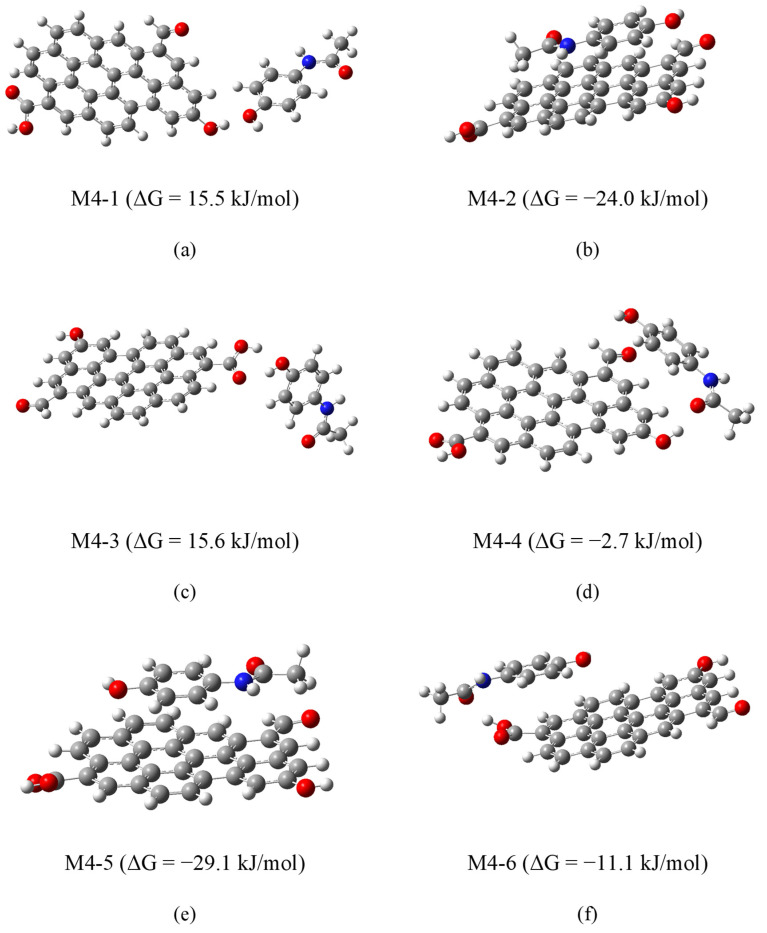
Optimized geometries of APAP with oxidized graphene in models M4−1 (**a**), M4−2 (**b**), M4−3 (**c**), M4−4 (**d**), M4−5 (**e**) and M4−6 (**f**), with corresponding ΔG values.

To isolate the role of dipolar interactions, additional models (M4−1 to M4−6) were constructed in which APAP functional groups (hydroxyl or acetamide) were positioned near oxygenated groups on graphene (hydroxyl, carbonyl, or carboxyl), intentionally avoiding initial π–π stacking. During optimization, some systems (M4−2, M4−5) evolved into π–π stacked conformations, indicating a thermodynamic preference for these interactions. This can be better appreciated in Figure 12. The APAP molecule is initially facing a graphene’s carbonyl group (model M4−5), yet as geometry optimization develops, it keeps moving to find the most stable position located in a parallel plane above the graphene model. Others (M4−1, M4−3) retained dipolar contact but exhibited positive or near-zero ΔG values, suggesting less favorable adsorption. Finally, in the other two cases (M4−4 y M4−6), an intermediate situation was reached. Figure 13 shows the optimized geometries for all these models.

These results suggest that although dipole interactions may contribute to adsorption under certain conditions, the dominant interaction mechanism for APAP on carbonaceous materials is π–π stacking. The relatively small energy differences among various binding sites further support the experimental observation that surface structure and chemical homogeneity critically influence adsorption behavior.

Some published results on the adsorption of APAP with different adsorbents are shown for comparison in Table 10. The results obtained when calculating the capacity of the monolayer according to the Langmuir model are shown, as well as experimental data.

Activated carbons are usually notable for their high adsorption capacity compared to other materials. MH carbon has a capacity comparable to the best adsorbents in the table. It should be noted that the adsorption conditions used are highly variable, so this comparison should be made with some caution.

A more comprehensive list can be found in [63]. The result obtained by MH (311.5 mg/g) is comparable to the best results in the cited article, and those that exceed this figure are, for the most part, results obtained at a more acidic pH than the range used in this manuscript.

## 3. Future Perspectives

The modification of adsorbents using hydrothermal methods is a new methodology that, to our knowledge, has only been tested by our research group [41,42,43,44], so there is little experience in its use in research and none in industrial processes, unlike what happens with the preparation of adsorbents using the hydrothermal method. According to the results we have obtained, its mechanism of operation is similar to that of other adsorbents such as commercial activated carbon. In most of the cases we studied, a more significant improvement over the initial activated carbon is obtained when metal cations are adsorbed than when organic contaminants are removed. However, as shown in this article, there are also cases in which the adsorption of organic compounds is improved. This is an issue that will be resolved with further research to determine when it is appropriate to apply this technology.

In real-world situations, the presence of other contaminants, such as natural organic matter (NOM), must be considered. Similar adsorbents, such as activated carbons, have been used for the removal of NOM [64]. This suggests that in real wastewater, there is competition between NOM and APAP for adsorption sites. This aspect is particularly important given that most NOM comprises low-polarity organic compounds. However, it should be noted that some pre-treatments, such as coagulation, can remove part of the NOM and avoid some of this competition, leaving adsorption to remove more recalcitrant compounds.

As for its industrial implementation, there should be no major difficulties as it is a simple process. To prepare these materials industrially, a third process (hydrothermal treatment) would have to be added to the two that are common in the manufacture of activated carbon (carbonization and activation). Considering that no reagents are used, as can be the case in activation, and that the working temperature is much lower tan in the other steps, it is expected that this third stage will not be costly and that the improvement obtained will offset this expense. The environmental aspect must also be taken into account: better performance means less production is needed, and since the raw material is usually biomass, there will be less demand for it, which could help combat deforestation in some parts of the world. As for adsorbent regeneration, it is a matter of doubtful use because it is not always cost-effective to regenerate an adsorbent, as it depends on numerous factors. In this regard, hydrothermal treatment could be an option worth considering, as it does not require expensive reagents (only water) and the temperatures required are not as high as in thermal regeneration.

As shown in Table 10 and further detailed in reference [63], MH has a comparatively high adsorption capacity and is also a low-cost material, especially when compared to others such as MOFs or CNTs, which do not improve performance enough to justify their higher cost.

## 4. Materials and Methods

### 4.1. Materials

Acetaminophen was purchased from Sigma-Aldrich (Merck Group, Darmstadt, Germany). Extra-pure commercial activated carbon granular 1.5 mm (Ref. 1.02514) was acquired also from Merck. Distilled water was used as a solvent in all determinations.

### 4.2. Synthesis of the Hydrothermal Carbon

#### MH Preparation

The commercial carbon material M (4 g) was treated with distilled water (50 mL) in a Teflon^®^ vessel reinforced with a duraluminium outer jacket at a temperature of 180 °C during 20 h. These conditions were selected based on previous studies [43,44,45] and the conditions that had led to improvements in the prepared adsorbents. In these studies and other unpublished tests conducted in our laboratory, we observed that materials prepared over prolonged periods (20 h) adsorb organic compounds better than those prepared over shorter periods (10 or 15 h). In terms of temperature, we were unable to obtain good adsorbents below 160 °C, and above 200 °C, we did not observe any appreciable differences within the limits tested. Given that temperature does not appear to influence adsorption capacity between 160 and 200 °C, we selected the average temperature of 180 °C. The content was filtered in a funnel with a filter plate and washed with 200 mL of distilled water. Then, it was dried in an oven at 110 °C for 24 h.

### 4.3. Characterization of the Activated Carbons

Textural characterization was performed by N_2_ adsorption isotherms at 77 K in an Anton Paar GmbH (Graz, Austria) Quantachrome Quadrasorb Evo equipment with prior degassing at 200 °C for 24 h, and the specific surface area was calculated by applying the BET method [65]. Elemental analysis (C, H, N, S, O) was performed using a Leco (St. Joseph, MI, USA) CHMS-932 elemental analyzer. C, H, N, and S were analyzed, and the difference was assigned to ash and oxygen content. Ash quantification was carried out in a Netzsch (Selb, Germany) STA 449 F3 Jupiter thermobalance (see heating program in Table S1). The point-of-zero-charge (PZC) values were determined using the method proposed by Valente Nabais and Carrott [66]. A Hitachi (Tokyo, Japan) field emission scanning electron microscope (SEM) S-4800 and a FEI (Hillsboro, OR, USA) field emission scanning electron microscope (SEM) Quanta 3D FEG, were used to explore the surface morphological characteristics of all samples. The sample analysis was performed under high-vacuum conditions. X-Ray photoelectron spectra (XPS) were recorded on a SPECS (Zürich, Switzerland) FlexPS-ARPES-E SPECS instrument using monochromatic Al Kα radiation at 1486.68 eV. Fourier-transform infrared spectra were recorded in a Bruker (Billerica, MA, USA) Vertex70 spectrometer in the 4000–400 cm^−1^ range, with a DLaTGS detector and a 0.4 cm^−1^ spectral resolution. A KBr tablet was prepared with 1/250 dilution. X-ray diffractograms were registered in a Bruker (Billerica, MA, USA) D8 Advance.

### 4.4. Adsorption Experiments

#### 4.4.1. Adsorption Kinetics

A total of 200 mL of a 100 mg/L acetaminophen (APAP) solution at pH 5, 6, and 7 was placed in Erlenmeyer flasks, the pH was adjusted by adding dropwise 0.01M HCl and NaOH buffer solutions, and then 200 mg of Merck (M) or Merck hydrothermal (MH) carbon was added. The flasks were placed in a constant-shaking bath at 30 °C and 40 °C, where 3 mL of each sample was taken at 5, 10, 20, 30, 60, 120, 180, 240, 300, 360, 420, 480, and 1440 min.

#### 4.4.2. Adsorption Isotherm

40 mL solutions containing 200, 300, 400, 500, and 600 mg/L of APAP at pH 5, 6, and 7 were placed in Erlenmeyer flasks, followed by the addition of 40 mg of adsorbent. The solutions were stirred for 24 h at 30 °C and 40 °C. The studies were performed in triplicate. After this time, an aliquot of the APAP solution was taken after 24 h, and the residual concentration was quantified.

The adsorption capacity of the materials was determined using Equation (5).

(5) $$q_{e}=\frac{\left(C_{i}-C_{f}\right)V}{m}$$

where *q_e_* is the adsorption capacity at 24 h (mg/g), *C_i_* is the initial concentration of APAP (mg/L), *C_f_* is the concentration of APAP at 24 h (mg/L), *V* is the volume of APAP placed in the tests (L), and *m* is the mass of adsorbent placed during the experiments (g).

#### 4.4.3. Analytical Determinations

Samples were analyzed using a UV-Vis spectrophotometer Shimadzu (Kyoto, Japan) at a wavelength of 249 nm.

#### 4.4.4. Computational Details

The computational study was performed using the Gaussian16 (Revision A.03) package [67] with the PW6B95D3/Def2SVP DFT level of theory. The calculations were carried out in water (SMD model) [68].

The thermodynamic parameters for the two-molecule models were calculated by subtracting the calculated data of the individual molecules from the joint model result.

To study which are the most important interactions between APAP and graphene we have used NBO Analysis. This method transforms the wave functions used in the DFT calculation into localized forms (bonds and orbitals). It is often used to determine the Lewis structure of a compound but, in our case, we have used the second-order perturbation theory to calculate the donor–acceptor interactions and estimate the energetic importance of each one.

## 5. Conclusions

Hydrothermal modification of commercial activated carbon significantly enhanced its physicochemical properties by increasing carbon and nitrogen content, reducing surface oxygen functionalities, and improving micro- and mesoporosity. The modified material (MH) exhibited superior adsorption performance for acetaminophen (APAP), achieving up to 94.3% removal under optimal conditions. Adsorption kinetics were best described by the pseudo-second-order model, while equilibrium data indicated that MH follows the Langmuir isotherm and M aligns more closely with the Freundlich model, reflecting differences in surface homogeneity. The adsorption process was only slightly favored at lower pH and higher temperatures. Density functional theory (DFT) calculations supported these findings, revealing that π–π stacking interactions dominate the adsorption mechanism and are strongest on non-oxidized carbon surfaces. Together, the experimental and computational results demonstrate that hydrothermal treatment is a promising and scalable strategy for enhancing the performance of activated carbon in the removal of pharmaceutical contaminants from water.

## Figures and Tables

**Table 1:** Proximate analysis (dry basis, wt%) of the carbon materials.

Sample	Fixed Carbon (%)	Volatile Matter (%)	Ash (%)
M	92.62	3.41	3.97
MH	90.78	5.13	4.09

**Table 2:** Elemental composition of the organic fraction (wt%).

Sample	C (%)	H (%)	N (%)	S (%)	O ^a^ (%)
M	81.54	2.12	0.77	0.71	14.86
MH	92.17	0.81	1.33	0.78	4.90

^a^ Oxygen content determined by difference.

**Table 3:** Surface elemental composition (atomic %) by XPS.

Sample	C 1s (%)	O 1s (%)	N 1s (%)	S 2p (%)	Si 2p (%)	Al 2p (%)
M	94.05	5.01	n.d.	0.13	0.61	0.20
MH	94.22	4.12	0.78	0.30	0.58	n.d.

**Table 4:** Surface elemental composition (wt%) by XPS.

Sample	C 1s	O 1s	N 1s	S 2p	Si 2p	Al 2p
M	91.36	6.48	0.00	0.34	1.39	0.44
MH	91.68	5.34	0.88	0.78	1.31	0.00

**Table 5:** Components of the C 1s peak obtained by deconvolution.

	B.E., eV (%)		
M	284.8 (52.75%)	285.7 (23.95%)	288.6 (23.3%)
MH	284.8 (74.69%)	286.5 (16.2%)	289.6 (9.11%)

**Table 6:** Textural properties from N_2_ isotherms.

	S_BET_	V_DR_	V_me_	V_total_
	m^2/^g	cm^3/^g	cm^3^/g	cm^3^/g
M	839.10	0.336	0.103	0.448
MH	808.26	0.384	0.130	0.533
model	BET	DR	DFT	

**Table 7:** Kinetic parameters for APAP adsorption on M and MH.

		M						MH					
		30 °C			40 °C			30 °C			40 °C		
		pH 5	pH 6	pH 7	pH 5	pH 6	pH 7	pH 5	pH 6	pH 7	pH 5	pH 6	pH 7
P-first order	q_e_	28.65	28.44	29.78	32.34	28.99	31.06	29.15	29.64	30.01	28.67	28.53	79.97
	*k*	0.0981	0.1004	0.0693	0.0702	0.0797	0.0769	0.0785	0.0758	0.0721	0.0864	0.0797	0.0267
	R^2^	0.7293	0.7393	0.6019	0.6139	0.6339	0.6599	0.5487	0.5391	0.5202	0.5860	0.5446	0.4169
P-second order	q_e_	34.01	33.33	29.41	34.25	34.13	31.95	51.02	50.51	49.50	52.63	51.55	50.51
	*k*	0.1882	0.1882	0.2058	0.1960	0.2067	0.1781	0.2489	0.2456	0.2608	0.2413	0.2640	0.2623
	R^2^	0.9976	0.9975	0.9975	0.9979	0.9983	0.9937	0.9995	0.9994	0.9996	0.9995	0.9997	0.9995
q_e_ exp		35.76	35.02	34.46	39.65	37.8	35.95	55.2	55.02	54.65	55.76	55.57	55.39

**Table 8:** Isotherm model parameters for APAP adsorption.

			Langmuir			Freundlich		
T, °C	Carbon	pH	R^2^	*S_m_*	*K_L_*	R^2^	*K_F_*	*n*
30	M	7	0.9615	298.5	1.08 × 10^−3^	0.9930	1.96 × 10^−3^	0.4574
30	MH	7	0.9945	307.7	1.81 × 10^−3^	0.9184	2.13 × 10^−6^	0.3125
40	M	7	0.9654	302.1	1.01 × 10^−3^	0.9960	1.57 × 10^−3^	0.4507
40	MH	7	0.9952	309.6	1.62 × 10^−3^	0.9168	1.28 × 10^−6^	0.3050
30	M	6	0.9670	296.7	1.02 × 10^−3^	0.9903	1.65 × 10^−3^	0.4521
30	MH	6	0.9930	308.6	1.70 × 10^−4^	0.9100	1.64 × 10^−6^	0.3086
40	M	6	0.9676	294.1	9.25 × 10^−4^	0.9915	7.96 × 10^−4^	0.4276
40	MH	6	0.9951	311.5	1.49 × 10^−4^	0.8982	1.22 × 10^−6^	0.3055
30	M	5	0.9729	302.1	9.54 × 10^−4^	0.9918	1.60 × 10^−3^	0.4538
30	MH	5	0.9931	308.6	1.55 × 10^−4^	0.8881	1.87 × 10^−6^	0.3125
40	M	5	0.9732	295.0	8.43 × 10^−4^	0.9950	5.28 × 10^−4^	0.4162
40	MH	5	0.9956	311.5	1.35 × 10^−4^	0.8766	1.21 × 10^−6^	0.3074

**Table 9:** Influence of initial APAP concentration on adsorption performance.

[APAP]	Material	Temperature (°C)	Adsorption Capacity (mg/g)			Removal Efficiency (%)		
			pH 5	pH 6	pH 7	pH 5	pH 6	pH 7
100 mg/L	M	30	79.44	78.70	78.15	75.30	74.60	74.10
		40	83.33	81.48	79.63	79.00	77.30	75.50
	MH	30	98.89	98.70	98.33	93.80	93.60	93.30
		40	99.44	99.26	99.07	94.30	94.10	94.00
500 mg/L	M	30	242.35	238.15	236.98	46.40	45.60	45.40
		40	244.63	240.74	239.88	46.90	46.10	45.90
	MH	30	300.00	297.84	295.19	57.50	57.00	56.50
		40	302.04	300.62	297.90	57.80	57.60	57.10

**Table 10:** APAP adsorption capacity of various adsorbents.

Amount of Absorbent g/L	APAP Concentration mg/L	q_e_/S_m_ mg/g	Adsorbent	Ref.
0.30	120	217 ^2^	Fly ash (K_2_CO_3_ activated)	[53]
0.30–0.60	120–480	270 ^1^	Fly ash (K_2_CO_3_ activated)	[53]
0.40	200	284 ^2^	KOH activated biochar	[54]
0.40	25–800	391.0 ^1^	KOH activated biochar	[54]
4.00	1000	29.3 ^1^	Activated granular carbon	[55]
0.50	50	1.091 ^1^	Biosorbent (spiky green horse-chestnut shell)	[56]
0.25–2.0	5–200	17.5 ^1^	H_3_PO_4_ activated carbon	[57]
1	2–250	114.35 ^1^	Ionic Liquid on mesoporous silica	[58]
5–120	40	13.92 ^2^	Ionic Liquid on mesoporous silica	[58]
0.5	10–100	58.80 ^1^	H_3_PO_4_ activated carbon	[59]
1.0	100	22.93 ^2^	H_3_PO_4_ activated carbon	[59]
0.60	20–150	213.84 ^1^	MgO/aminated β-cyclodextrin	[60]
5.0	10.5	29.44 ^2^	Porous graphene	[40]
5.0	0.1–21	47.85 ^1^	Porous graphene	[40]
---	3–50	5.963 ^1^	MOF	[61]
0.05–0.1	100	231.5 ^3^	SWCNT	[62]
0.05–0.1	100	81.6 ^3^	MWCNT	[62]
1.0	200–600	311.5 ^1^	MH	This work
1.0	500	300.6 ^2^	MH	This work

^1^ S_m_ Langmuir. ^2^ Experimental data. ^3^ Polanyi–Manes model.

## Data Availability

The data will be provided upon request to the authors.

## Supplementary Materials

The following supporting information can be downloaded at: https://www.mdpi.com/article/10.3390/molecules30214295/s1, Figure S1. SEM images of M; Figure S2. SEM images of MH; Figure S3. Models of APAP (M1), graphene (M2a) and oxidized graphene (M2b); Figure S4. Kinetic plots of APAP adsorption at different pHs and temperatures; Figure S5. X-ray diffractograms of M and MH; Table S1. Heating program.
