# Supplementary material for: Hydrothermal Modification of Activated Carbon Enhances Acetaminophen Adsorption: Experimental and Computational Evidence of π–π Interaction Dominance
Source: Molecules. 2025 Nov 5;30(21):4295. doi: 10.3390/molecules30214295 (PMC12609994; doi:10.3390/molecules30214295)
Supplement: Supplementary file 1 [file molecules-30-04295-s001.zip › molecules-3897611-supplementary.pdf]

Supplementary Information

# Hydrothermal Modification of Activated Carbon Enhances Acetaminophen Adsorption: Experimental and Computational Evidence of π–π Interaction Dominance

**Astrid G. Cortés-Cruz [1], Marta Adame-Pereira [2], Carlos J. Durán-Valle [2,3,*] and Ignacio M. López-Coca [2,4]**

[1] Área Académica de Química, Universidad Autónoma del Estado de Hidalgo, Mineral de la Reforma C.P. 42184, Mexico; co483027@uaeh.edu.mx
[2] Departamento de Química Orgánica e Inorgánica, Universidad de Extremadura, Avda. del Elvas, s/n, 06006 Badajoz, Spain; martaap@unex.es (M.A.-P.)
[3] Instituto Universitario del Agua, el Cambio Climático y la Sostenibilidad, Universidad de Extremadura, Avda. del Elvas, s/n, 06006 Badajoz, Spain
[4] Instituto Universitario para el Desarrollo Territorial Sostenible, Universidad de Extremadura, Avda. de la Universidad, s/n, 10003 Cáceres, Spain
* Correspondence: carlosdv@unex.es

*Scanning Electron Microscopy*

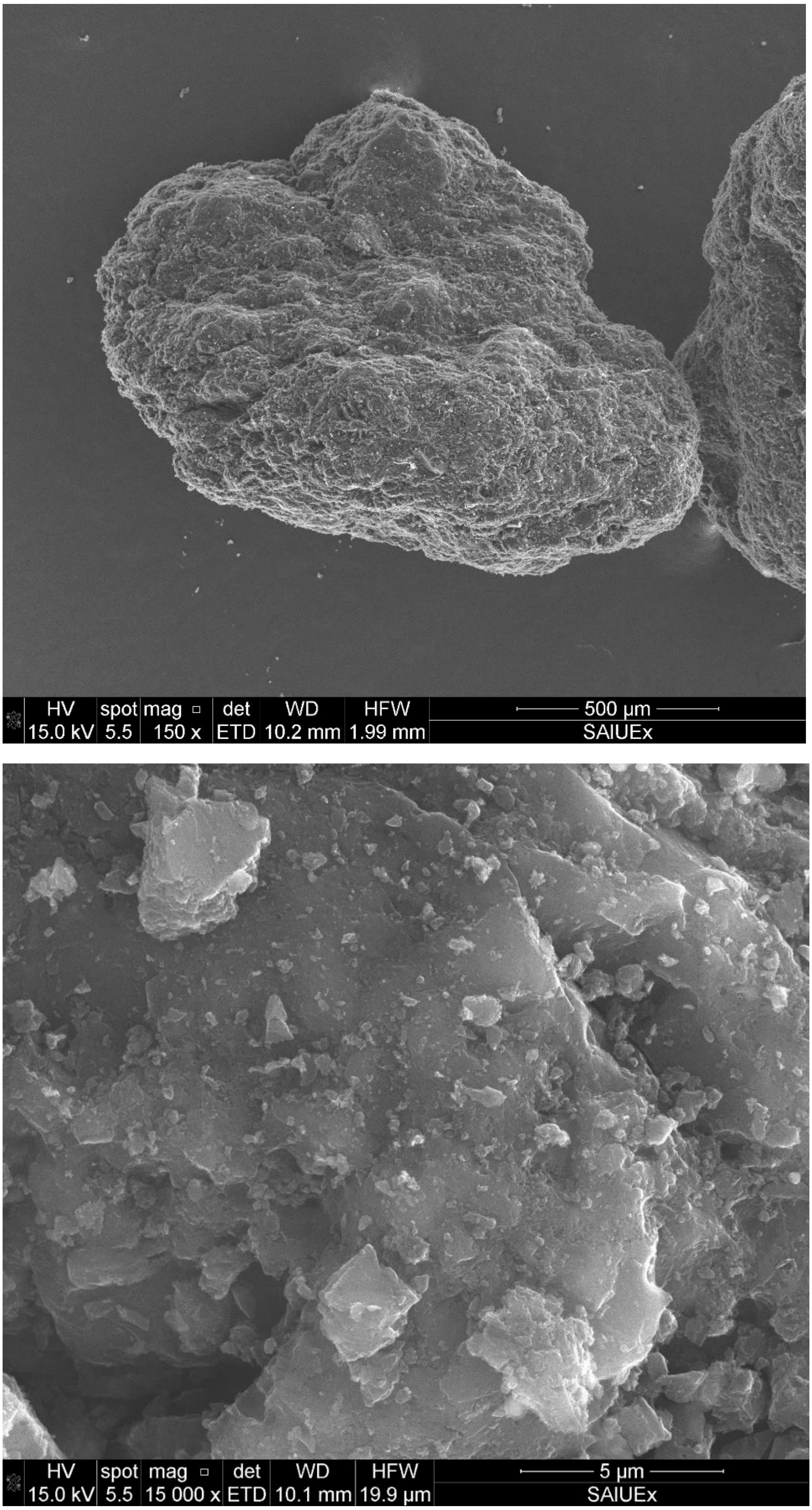


Figure S1. SEM images of M.

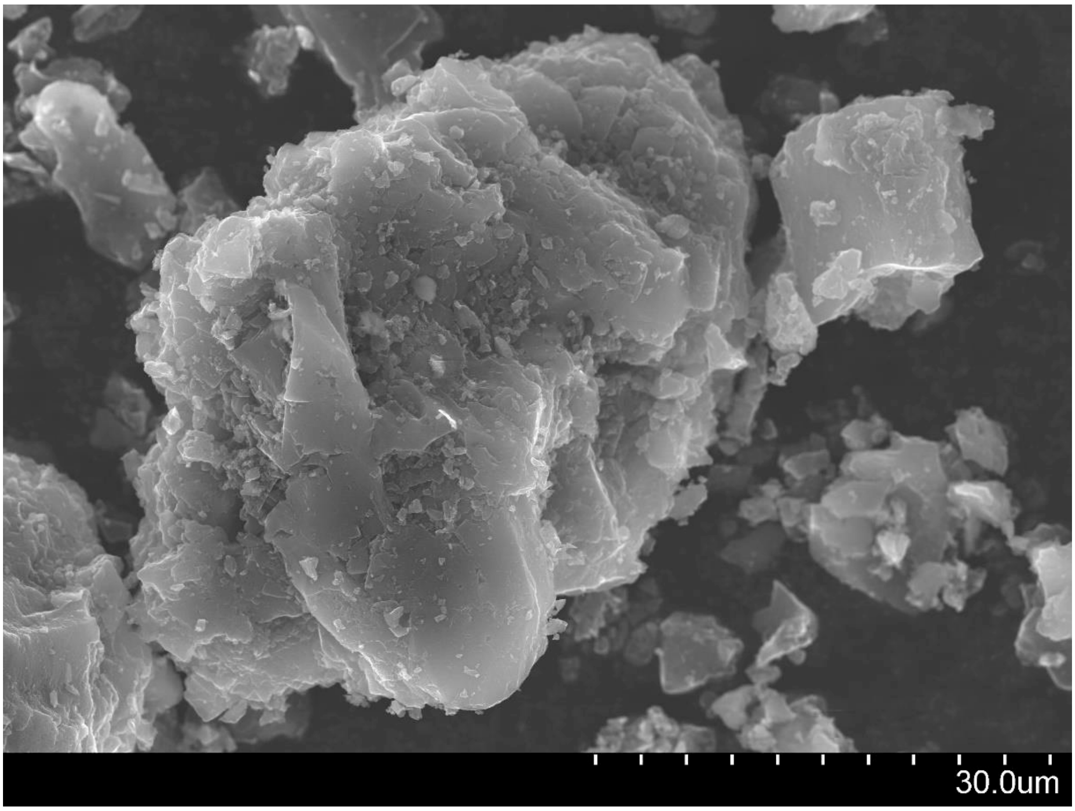


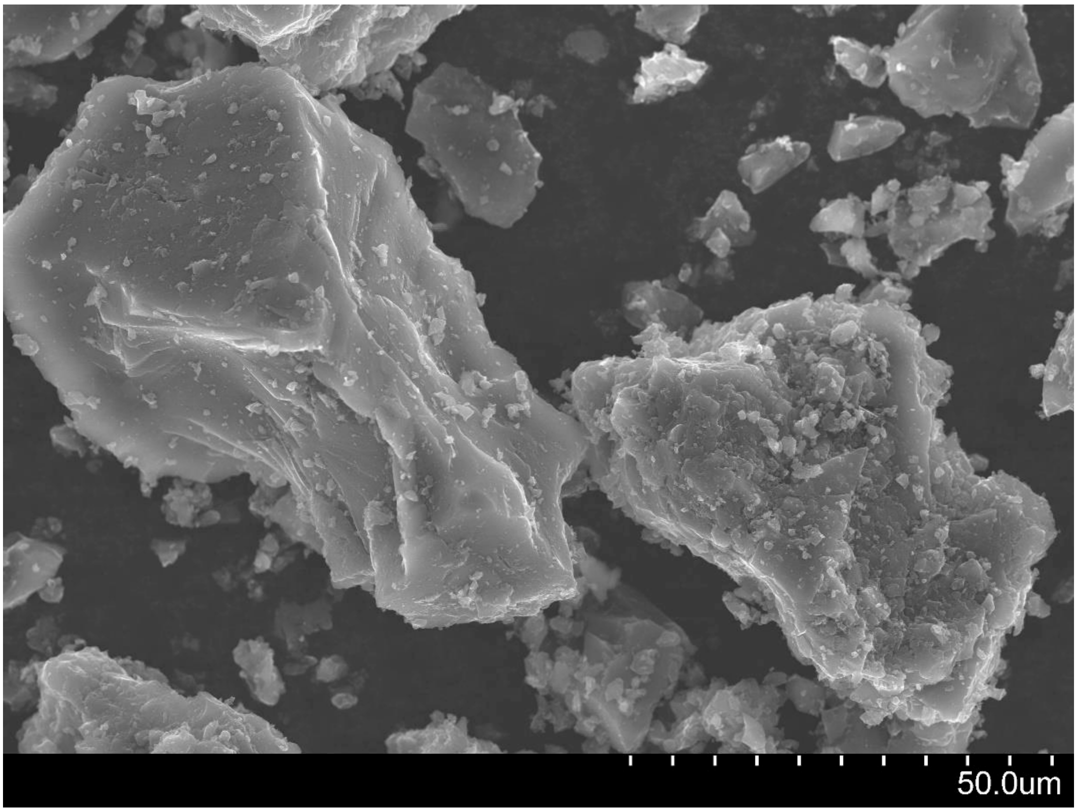


Figure S2. SEM images of MH.

*Some computational models*

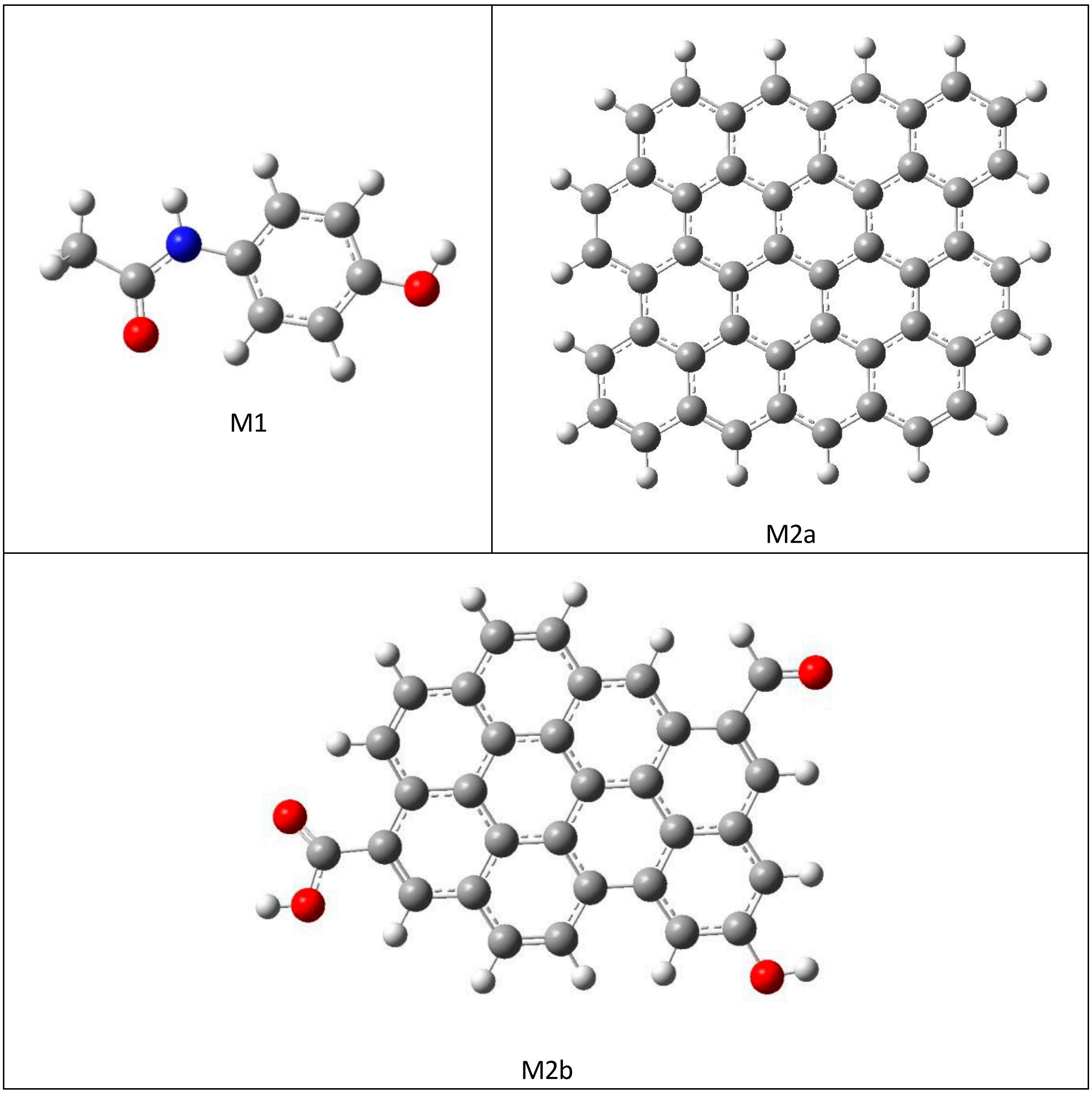


Figure S3. Models of APAP (M1), graphene (M2a) and oxidized graphene (M2b).

*Kinetics*

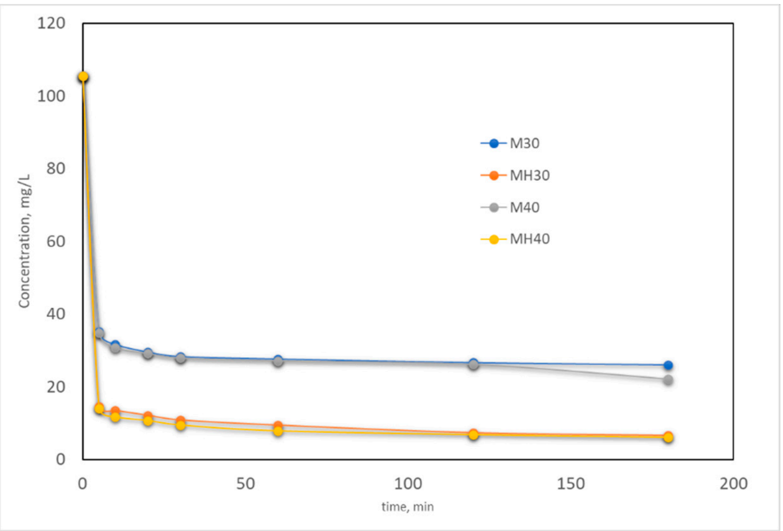


(a) pH 5

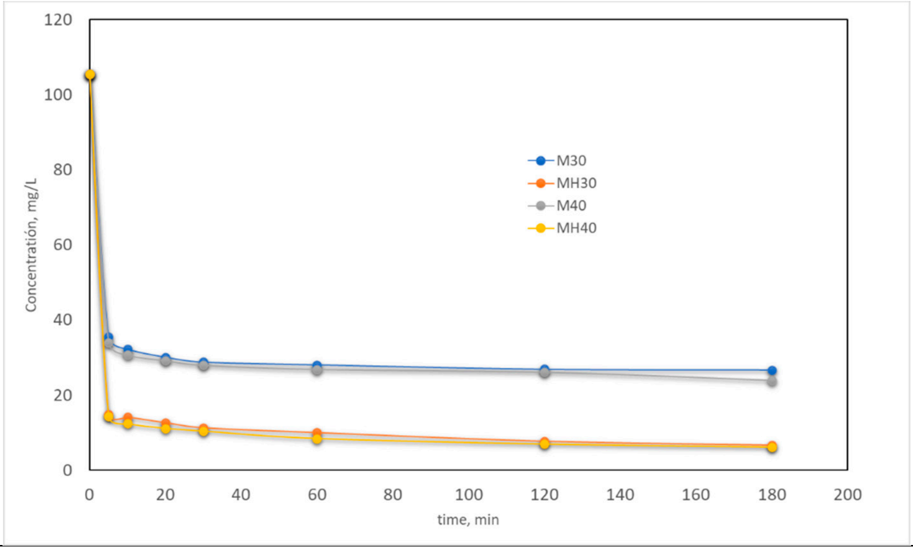


(b) pH 6

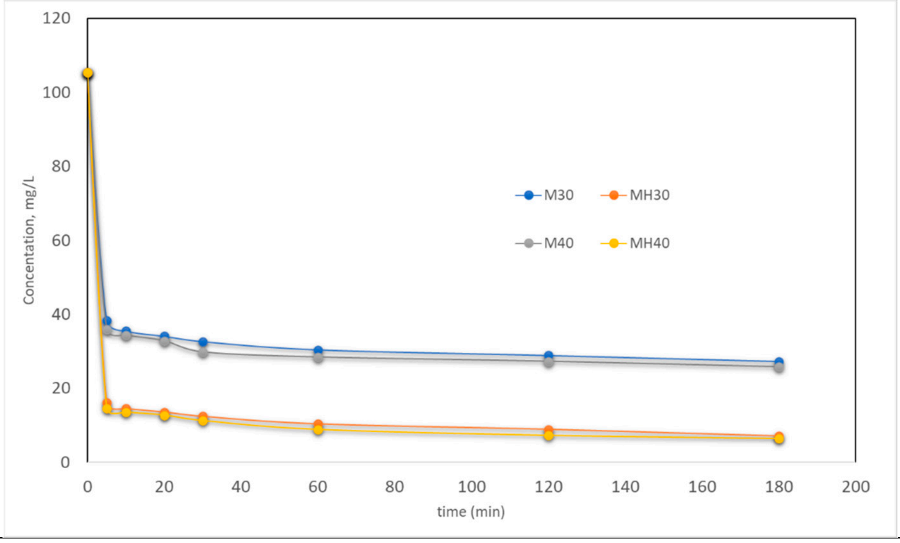


(c) pH 7

**Figure S4.** Kinetic plots of APAP adsorption at different pHs and temperatures.

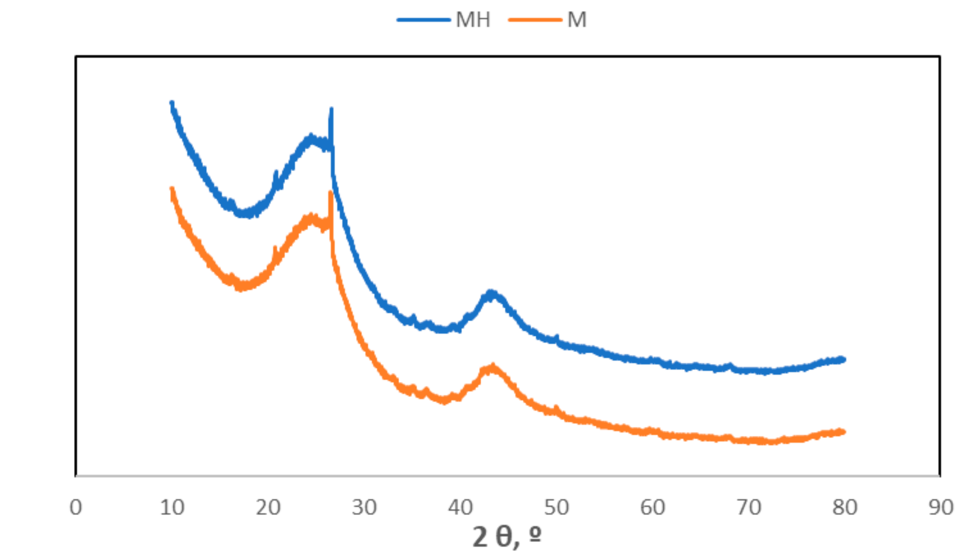


**Figure S5**. X-ray diffractograms of M and MH.

*Proximate analysis*

Table S1. Heating program

| $T_i$ ºC | $T_f$ ºC | Heating rate, ºC $min^{-1}$ | It, min | Atmosphere | Gas flow, mL $min^{-1}$ | Step |
|---|---|---|---|---|---|---|
| 40 | 40 | | 30 | Ar | 100 | Stabilization |
| 40 | 105 | 10 | | Ar | 100 | Humidity measurement |
| 105 | 105 | | 10 | Ar | 100 | |
| 105 | 900 | 30 | | Ar | 100 | V. M. measurement |
| 900 | 900 | | 7 | Ar | 100 | |
| 900 | 900 | | 90 | $Ar/O_2$, 80:20 | 100 | Measurement of F.C. and ash |

$T_i$, initial temperatura; $T_f$, final temperatura; it, isothermal time
